# Enhanced effectiveness of oil dispersants in destabilizing water-in-oil emulsions

**DOI:** 10.1371/journal.pone.0222460

**Published:** 2019-09-16

**Authors:** Gerald F. John, Joel S. Hayworth

**Affiliations:** 1 Science and Technology Department, Bryant University, Smithfield, Rhode Island, United States of America; 2 Civil Engineering Department, Auburn University, Auburn, Alabama, United States of America; Chinese Academy of Forestry, CHINA

## Abstract

Oil impacting the northern Gulf of Mexico shoreline from the 2010 *Deepwater Horizon* accident was predominantly in the form of water-in-oil emulsions (WOE), a chemically weathered, highly viscous, neutrally buoyant material. Once formed, WOE are extremely difficult to destabilize. Commercially-available oil dispersants are largely ineffective de-emulsifiers as a result of the inability of dispersant surfactants to displace asphaltenes stabilizing the oil-water interface. This study investigated the effectiveness of the commercially-available dispersant Corexit 9500A, modified to enhance its polar fraction, in destabilizing WOE. Results suggest that Corexit modified to include between 20–60% fractional amount of either polar additive (1-octanol or hexylamine) will produce a modest increase in WOE instability, with a Corexit to hexylamine ratio of approximately 80/20 providing the most effective enhanced destabilization. Results support the hypothesis that modifying the fraction of polar constituents in commercial dispersants will increase asphaltene solubility, decrease oil-water interface stability, and enhance WOE instability.

## Introduction

The explosion and sinking of the *Deepwater Horizon* (DWH) drilling platform, while exploring for oil on the Macondo Prospect (MC252) in the Gulf of Mexico (GOM), resulted in the discharge of an estimated 785 million liters of crude oil into the GOM between April 20, 2010 and July 15, 2010 [1, 2]. Of this estimated total volume, approximately 611 million liters have been accounted for through direct recovery, evaporation and dissolution, natural and chemical dispersion, and burning or skimming [1]. Two recent studies [3, 4] suggest that between 14–113 million liters of MC252 oil may be deposited on the deep sea floor in the vicinity of the MC252 well, in an area ranging from 3,200–24,000 km^2^. It is likely that a large fraction of the remaining 62–161 million liters of oil washed onto northern GOM shorelines in Florida, Alabama, Mississippi, and Louisiana [5–8].

MC252 oil impacting northern GOM shorelines was predominantly in the form of water-in-oil emulsions (WOE), a chemically weathered, highly viscous, neutrally buoyant material [9, 10]. WOE form when raw crude oil is released in aquatic systems, is weathered over time (through loss of volatile constituents, photodegradation, and biodegradation), and is mechanically mixed with water primarily by wind and wave-induced shear stress [11, 12]. Once formed, WOE are extremely difficult to destabilize, even when treated with dispersants [9, 13]. Stable WOE contains 50%-80% water by volume, resulting in a considerable increase in the volume of oil-related material requiring cleanup. Additionally, the density and viscosity of WOE increases relative to the density and viscosity of the original raw crude oil [14], which has important implications with respect to the physical, chemical, and transport behavior of the emulsion. Generally, WOE will extend deeper into the water column, will evaporate and spread more slowly, and will be less influenced by wind in their transport behavior relative to non-emulsified oil [14].

Previous studies have shown that stable WOE form as a result of surfactant-like behavior of asphaltenes within the crude oil. Asphaltenes constitute the non-volatile, higher molecular weight fraction of crude oil. Importantly, asphaltenes are more soluble in crude oil when associated with polar resins and other polar constituents of crude oil. It is thought that asphaltenes stabilize WOE by collecting at the water-oil interface as colloidal aggregates. These asphaltenic molecules are thought to aggregate through hydrogen bonding and proton/electron donor-acceptor interactions and are solvated on their edges primarily by polar resins. There is evidence that as WOE are forming, polar resin molecules (which initially serve to solvate asphaltene aggregates) are shed, leading to film stabilization. The end result is a cross-linked, three-dimensional, mechanically rigid, viscous film at the water droplet-oil interface which resists coalescence of water droplets dispersed in the emulsion [15, 16].

Exact oil dispersant formulations are proprietary, but all contain one or more nonionic or anionic surfactants. Cationic surfactants are not used in current dispersant formulations because of their potential toxicity to many organisms [17]. Studies directed at developing methods for destabilizing WOE using commercially-available oil dispersants as de-emulsifiers are limited. Fingas et al. [18] considered two oil dispersants (the Environment Canada dispersant Vytac DM, and a 60% Alcopol solution). Results indicated that the effectiveness of these dispersants at breaking the emulsion was highly dependent on the experimental methods used to conduct the tests and the dispersant used in the tests, with a considerable variation in dispersant-to-emulsion ratios ranging from 1:7000 to 1:250.

More recently, experiments conducted by Bureau of Ocean Energy Management, Regulation and Enforcement (BOEMRE) using Corexit 9527 and Corexit 9500A showed limited de-emulsification for WOE with viscosities of 2630 cPs or greater using dispersant-to-emulsion ratios as high as 1:20 [19]. Corexit 9500A (developed in the 1990’s) was the primary dispersant used in DWH response efforts, and together with Corexit 9527 (developed in the 1980’s) comprise the most common commercially available and used oil dispersants world-wide. In the U.S., Corexit 9527 and 9500A make up approximately 95% of all industry stockpiles. Corexit 9500A contains the same surfactants as Corexit 9527, but uses a different solvent mixture to reduce adverse health effects observed in first responders exposed to Corexit 9527. Additionally, the solvent mixture used in Corexit 9500A has been shown to be slightly more effective at dispersing high-viscosity oils than Corexit 9527 [20].

The relative ineffectiveness of commercially available oil dispersants to destabilize WOE is thought to be a result of the inability of the dispersant surfactants to displace asphaltenes stabilizing the oil-water interface. This observation, along with the observation that asphaltene solubility increases in oil when associated with polar resins and other polar constituents in the oil, suggests that modifying the composition and fraction of polar constituents in commercial dispersants may increase asphaltene solubility, decrease oil-water interface stability, and enhance the de-emulsification ability of these dispersants.

In this study, we investigate the effectiveness of the commercially-available dispersant Corexit 9500A, modified to enhance its polar fraction, in the destabilization of WOE. Experiments described here used artificial (laboratory-developed) WOE formed from laboratory-weathered Louisiana light sweet crude oil. This oil is the official surrogate of MC252 oil provided to researchers by the US Department of Interior [21]. Two polar additives to Corexit 9500A were considered (1-octanol and hexylamine) based on their mechanistic differences in destabilizing emulsions and their negligible environmental risk. [22, 23]. 1-octanol is a short‐chain alcohol, which breaks down intermolecular hydrogen bonds between asphaltene molecules, replacing them with alcohol‐asphaltene hydrogen bonds [22]. Wasan et al. [23] observed similar destabilizing effect for medium‐chain alcohols. Hexylamine disintegrates asphaltenes through interaction between the nitrogen group (base) and the acid groups present in the interfacial film, making this film more hydrophilic [22, 24].

## Materials and methods

### Raw crude oil, dispersant, and artificial seawater

**Fig 1:**
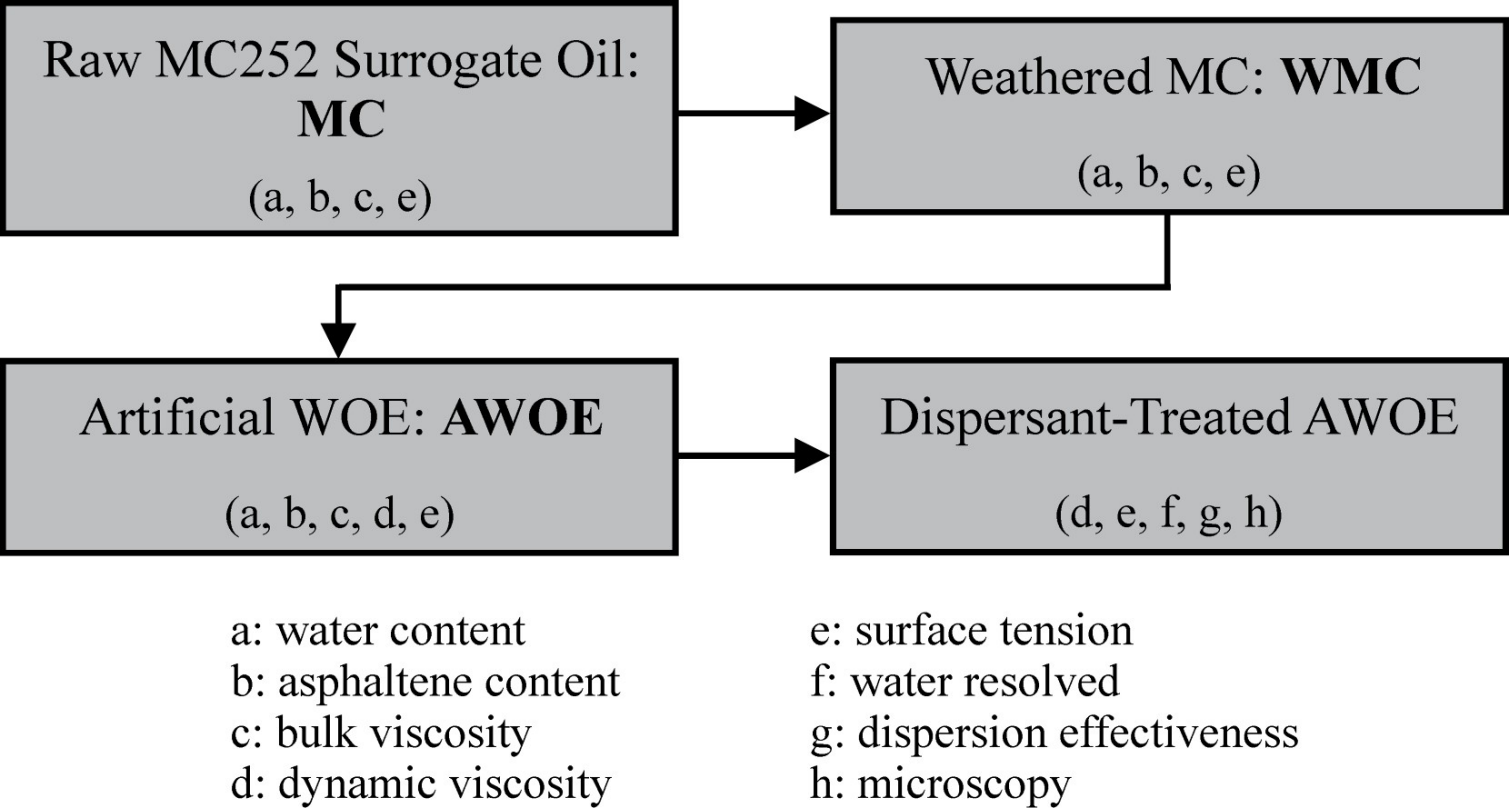
Summary of oil preparation, measurements, and experiments.

Louisiana light sweet crude oil (MC252 surrogate oil, referred to in this paper as MC) was supplied by AECOM (Fort Collins, CO, USA). Corexit 9500A (dispersant) was supplied by the National Oil Spill Response Test Facility (OHMSETT), NJ, USA. Hexylamine and 1-octanol were supplied by Sigma-Aldrich, USA. Xylenes and sodium chloride were supplied by VWR, USA. Corexit dispersant was modified by adding either hexylamine or 1-octanol to Corexit in the proportions of 20, 40, 60, 80 and 100% v/v (Table 1). Artificial seawater (ASW) was prepared by dissolving 33 g NaCl into 1L deionized water (3.3 wt%; pH approximately 7.5) [25]. A summary of oil preparation and measurements/experiments is shown in Fig 1.

**Table 1:** Dispersant mixtures used in study.

Dispersant Mixture	Corexit 9500A % (v/v)	Additive Fraction % (v/v)
C(100)	100	0
C/X (80/20)	80	20
C/X (60/40)	60	40
C/X (40/60)	40	60
C/X (20/80)	20	80
X (100)	0	100

C: Corexit; X: 1-octanol or hexylamine

### Weathered MC252 raw crude oil (WMC)

WMC was created by uniformly distributing a thin film of MC onto glass plates, and then placing these in boxes covered with glass plates. The boxes were well ventilated to prevent overheating of oil samples. The samples were exposed to sunlight from 10 am to 4 pm for 4 days (approximately 24 hours of sunlight), resulting in a mass reduction of 29 ± 5%.

### Artificial water-in-oil emulsion (AWOE)

**Fig 2:**
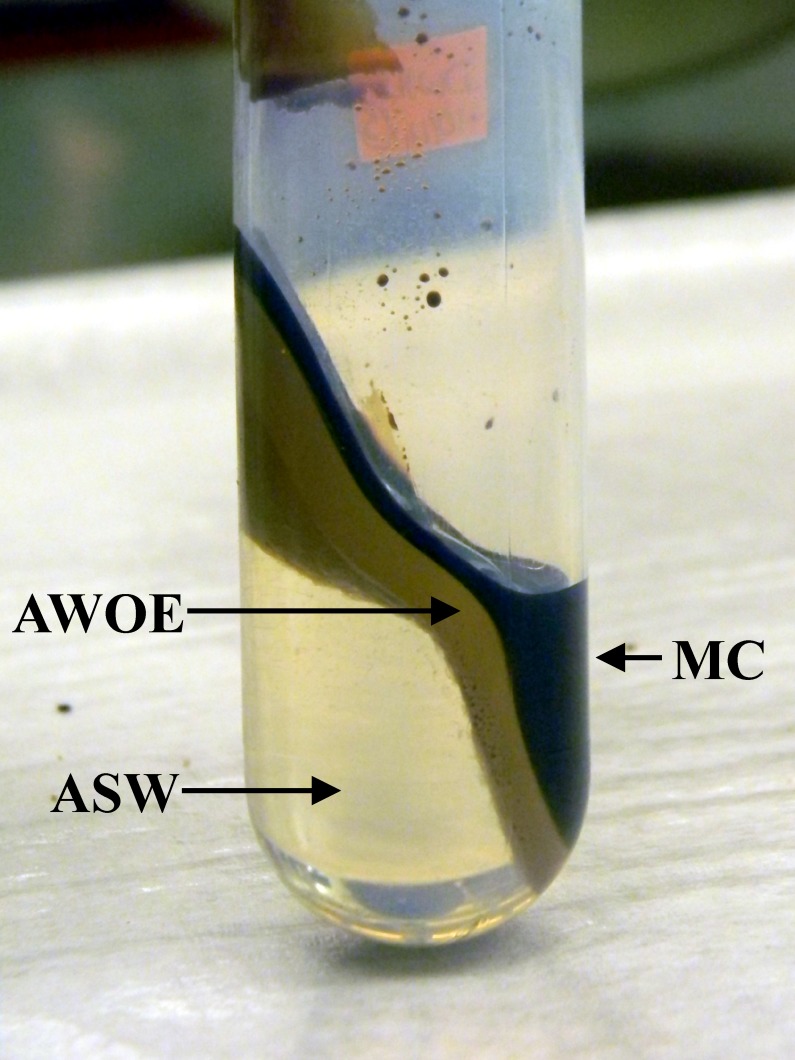
Preparation of AWOE from MC and ASW.

AWOE was prepared from WMC using a commercial blender [26]. WMC (15 g) and ASW (35 g) were placed in a commercial blender and mixed at maximum speed for 1 minute, after which oil/water mixture adhering to the blender container walls was scraped to the bottom of the blender container using rubber scraper. This process was repeated twice; the contents were then transferred to 50 mL centrifuge tubes and allowed to stand for 8 hours. The contents were then centrifuged at 15000 rpm for 1 hour, resulting in the formation of three distinct, separate layers (Fig 2): a top layer containing WMC, a bottom layer containing ASW, and a middle layer containing AWOE. AWOE prepared from several batches was combined and mixed to form a single composite sample used during this study.

### Water content

The water content of MC, WMC and AWOE was measured using the American Society for Testing and Materials (ASTM) method D4006 [27]. MC, WMC or AWOE (5–10 g) was placed in a 1 L round-bottomed flask and a solvent mixture of xylenes was added to make the total volume 400 mL. Heat was applied to the bottom of the flask to drive water into a trap; the mass percentage of water present in the sample was determined using:

$$\mathrm{Mass}\;(\%)=\frac{{(V}_{T}{‐V}_{B})}{M}100$$ (1)

where V_T_ is the volume of water in the trap, V_B_ is the volume of water in the solvent blank (xylenes), and M is the mass of the sample.

### Asphaltene content

The asphaltene content of MC and WMC was determined using ASTM method D3279 [28]. About 5–10 g of MC or WMC was placed in a 125 mL Erlenmeyer flask and 100 mL of n-hexane was added. The flask was fitted with a reflux condenser and heated at 45°C for 20 minutes. The contents were then passed through a glass-fiber filter, and the filter was dried to determine the asphaltene content of the sample. The asphaltene content of AWOE is equivalent to that of WMC; it was not directly measured because the presence of water in the AWOE negatively affected the filtering process.

### Bulk viscosity

The bulk viscosity of MC and WMC was measured using a Brookefield Viscometer (DV-II+) employing a spindle (size 21) and a small sample adapter. About 8 g of MC or WMC was placed in the small sample adapter, the spindle was then inserted into the adapter and the viscometer was turned on to measure the viscosity. The bulk viscosity of AWOE was measured using a TA AR2000e dynamic shear rheometer (DSR) following a previously established method [14]. Sample viscosity was measured using 25 mm stainless steel plates. An AWOE sample was placed between the stainless-steel plates, and the plate gap was adjusted to 0.5 mm. After the sample was thermally equilibrated (25°C for 2 minutes), AWOE bulk viscosity was measured. A stress sweep from 0.1 to 10 Pa at a frequency of 1 Hz was performed and an oscillating shear stress of 1 Pa was used for the viscosity measurement.

### ASW resolved from and retained by AWOE

A series of experiments were conducted to estimate the amount of ASW resolved from, and retained by AWOE after treatment with dispersant or modified dispersant. About 30 g (7.5 g oil equivalent) of AWOE was weighed into a 40 mL vial. 300 μL of dispersant or modified dispersant (1:25 dispersant or modified dispersant to oil ratio) was added and the contents were mixed using an end-to-end rotary mixer at 50 rpm for 12 hours. The contents were then allowed to settle for 12 hours. At the end of 12 hours of settling, for AWOE-dispersant and AWOE-modified dispersant below approximately 60% polar additive fraction, three different layers were formed: an upper layer contained WMC, a middle layer contained the fraction of AWOE remaining after dispersant treatment, and a bottom layer of resolved ASW. The top layer containing WMC was carefully removed. ASW was then carefully removed using a long stem Pasteur pipette, and its volume was measured to determine the amount of ASW resolved from AWOE after treatment with dispersant or modified dispersant. The middle layer (remaining AWOE) was collected for analysis of dynamic viscosity and microscopy. For AWOE-modified dispersant above approximately 60% polar additive fraction, very little to no separation between ASW, WMC, and AWOE occurred. The mixture remained a homogenous emulsion which macroscopically was more fluid (less viscous) than untreated AWOE or AWOE remaining after treatment with dispersant or modified dispersant below approximately 60% additive fraction.

### Dynamic viscosity

The dynamic viscosity of AWOE (untreated and treated with dispersant/modified dispersant) as a function of shear stress was measured by varying the oscillating shear stress of the DSR. The lowest oscillating shear stress was set at 1 Pa and was increased logarithmically at a frequency of 0.1 Hz. The oscillating shear stress was increased until the sample spilled out from between the DSR plates. For each step, the stress was applied for 30 s, with the difference in the oscillating stress and strain response being measured over two waveform periods.

### Surface tension

Surface tension of MC, WMC, and AWOE (untreated and treated with dispersant or modified dispersant) was measured with a Kyowa Interface Science surface tensiometer (DY-700) using the Wilhelmy plate method. Surface tension was measured as a surrogate for interfacial tension for two reasons: (1) the change in surface tension between MC, WMC, and AWOE could be compared, and (2) directly measuring interfacial tension within AWOE was not possible. MC, WMC, and untreated/treated AWOE were thermally equilibrated, placed in clean glass vessels and tested at room temperature (25°C). The Wilhelmy plate was rinsed and heat-cleaned with an alcohol lamp to remove any residual material before each measurement. At least 60 seconds were given for the Wilhelmy plate to cool down between measurements.

### Dispersion effectiveness

Dispersion effectiveness (DE) is a measure of the ability of a dispersant to disperse oil into the water-phase [25]. Following a DE experiment (described below), DE was determined by ultraviolet-visible spectrophotometric analysis (UV-Vis) of the water-phase for the presence of oil. For a given oil, dispersants having higher DE are considered more effective at dispersing oil into the water-phase. Although DE studies have been performed using various raw and weathered oil-dispersant combinations [25, 29, 30], to the best of our knowledge no studies have yet applied this approach to WOE-dispersant systems.

To generate a calibration curve (6 point) for UV-Vis analysis, oil standards were first prepared as in previous studies [25, 29] with certain modifications. Stock solutions of dispersant–oil mixtures in dichloromethane were prepared by adding 80 μL of Corexit to 2 g of oil (MC or WMC), and then adding 18 mL of dichloromethane. WMC was used as the AWOE oil standard, since AWOE was prepared from WMC. Specific volumes of the stock standard solution (20, 50, 100, 200, 500 and 1000 μL) were added to 30 mL of ASW in a 50 mL separatory funnel. The mass of oil for each calibration point was estimated based on density calculations. Liquid/liquid extraction was performed by adding 5 mL of dichloromethane. The liquid/liquid extraction process was performed three times; extracts were combined and adjusted to a final volume of 25 mL, and analyzed using a UV-Vis for absorbance at wavelengths 340, 370, and 400 nm [25]. The area under the absorbance vs. wavelength curve between wavelengths 340 and 400 nm was determined by applying the trapezoidal rule:

$$A=\frac{\left({\mathrm{Abs}}_{340}+{\mathrm{Abs}}_{370}\right)}{2}30+\frac{\left({\mathrm{Abs}}_{370}+{\mathrm{Abs}}_{400}\right)}{2}30$$ (2)

where A is the area under the curve. The mass of oil and its respective area were plotted to compute a calibration slope.

Dispersion effectiveness experiments followed the approach of Venosa and Holder [25]. A 150 mL baffled trypsinizing flask fitted with a PTFE stopcock at the bottom was used. 120 mL of ASW was added into the trypsinizing flask. About 100 mg of oil (MC, WMC, or AWOE) was then carefully added to the surface of the ASW. For AWOE, 100 mg oil equivalent (about 400 g of AWOE) was added to the ASW to normalize AWOE dispersant effectiveness measurements to those of MC and WMC. 4 μl of dispersant or modified dispersant was then carefully added to the surface of the oil. The ratio of dispersant or modified dispersant to oil was 1/25 (v/w) [25]. The baffled flask was placed on an orbital shaker at 200 stokes/minute for 10 minutes. Prior studies have shown that the baffled flask approach, combined with this orbital speed, is representative of moderately turbulent mixing in open water bodies [25, 31]. The contents were allowed to sit for 10 minutes to allow non-dispersed oil to return to the surface, 2 mL of subsurface ASW was drained from the stopcock and discarded, and then 30 mL of subsurface ASW was collected and transferred to a 50 mL separatory funnel. Liquid/liquid extraction was performed by adding 5 mL of dichloromethane. The liquid/liquid extraction process was performed three times. The extracts were combined and adjusted to a final volume of 25 mL, and analyzed using a UV-Vis for absorbance at wavelengths 340, 370, and 400 nm [25] (similar to the calibration measurement). The area under the absorbance vs. wavelength curve between wavelengths 340 and 400 nm was determined using (2). This area was then used to compute DE using:

$$\mathrm{DE}\;(\%)=\frac{\mathrm{Area}}{\mathrm{Calibration}\;\mathrm{slope}}\frac{V_{\mathrm{tw}}}{V_{\mathrm{ew}}}\frac{1}{M_{\mathrm{oil}}}100$$ (3)

where V_tw_ is the total volume of ASW, V_ew_ is the volume of ASW extracted, and M_oil_ is the mass of oil (MC, WMC, or AWOE) added to the flask.

### Microscopic analysis

About 10 μL of AWOE (untreated or treated with dispersant or modified dispersant) was placed on a glass slide and observed in both bright field and fluorescence mode using an Advanced Microscopy Group Model AMAFD1000 microscope.

## Results and discussion

Water and asphaltene content, bulk viscosity, and surface tension for MC, WMC, and untreated AWOE are given in Table 2, showing the increase resulting from weathering (MC to WMC) and the formation of water-in-oil emulsions (WMC to AWOE). As expected, MC and WMC water content was non-detectable. AWOE prepared from WMC and ASW was stable, with no loss of water content and no water droplet coalescence even after several weeks of storage. Of particular interest is the increase in AWOE surface tension and bulk viscosity relative to WMC: since only AWOE contains water and the asphaltene content of WMC and AWOE are the same, the increase in AWOE surface tension and bulk viscosity is primarily a result of the adhesive forces (interfacial tension) between WMC and ASW within the AWOE.

**Table 2:** Properties of materials not treated with dispersant or modified dispersant.

Parameter	MC	WMC	AWOE
Water content (v/m; %)	BD	BD	75 ± 5
Asphaltene content (%)	0.417 ± 0.007	1.1 ± 0.2	1.1 ± 0.2 *
Bulk Viscosity (cPs)	9.5 ± 0.5	217 ± 2	6760 ± 340
Surface Tension (mN/m)	19	21	36

BD: below detection

*: same as WMC

**Fig 3:**
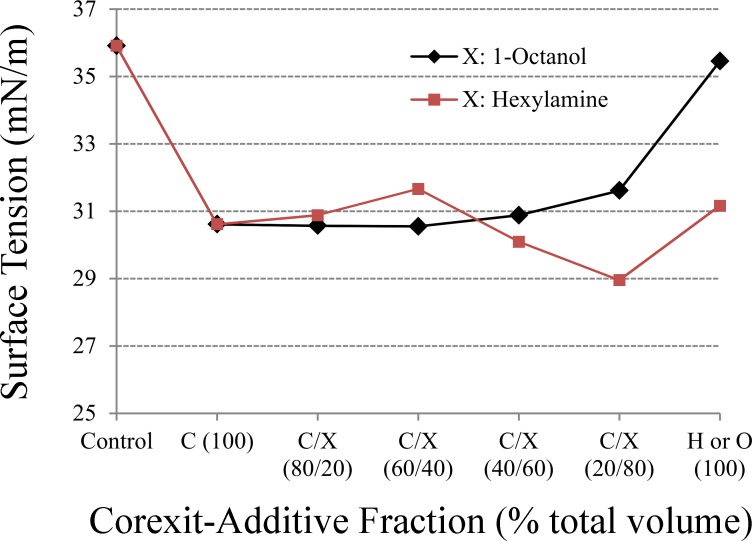
AWOE surface tension (surrogate for interfacial tension) as a function of dispersant/modified dispersant fractions (C: Corexit dispersant).

Surface tension (as a surrogate for interfacial tension) of AWOE as a function of dispersant or modified dispersant additive fraction is shown in Fig 3. Control values (no dispersant or modified dispersant) corresponds to the value given in Table 2. Fig 3 illustrates the decrease in AWOE interfacial tension resulting from the addition of surface active dispersant and dispersant additives. In general, lowering interfacial tension in liquid-liquid emulsions increases miscibility of the two liquids (through the accumulation of surface active polar molecules at liquid-liquid interfaces), a positive de-emulsification outcome. Fig 3 suggests that surfactants within the dispersant are the primary surface active compounds, since up to an additive fraction of approximately 40%; interfacial tension is essentially unchanged from the 100% dispersant condition (no polar additives). Fig 3 also indicates that 1-octanol is ineffective at lowering AWOE interfacial tension: as the fractional amount of 1-octanol increases, interfacial tension also increases, eventually returning to the control state when the additive is 100% 1-octanol. For hexylamine, additive fractions between 60%-80% slightly reduce AWOE interfacial tension below the 100% dispersant condition, and 100% hexylamine appears to be as effective as 100% dispersant with respect to lowering AWOE interfacial tension.

**Fig 4:**
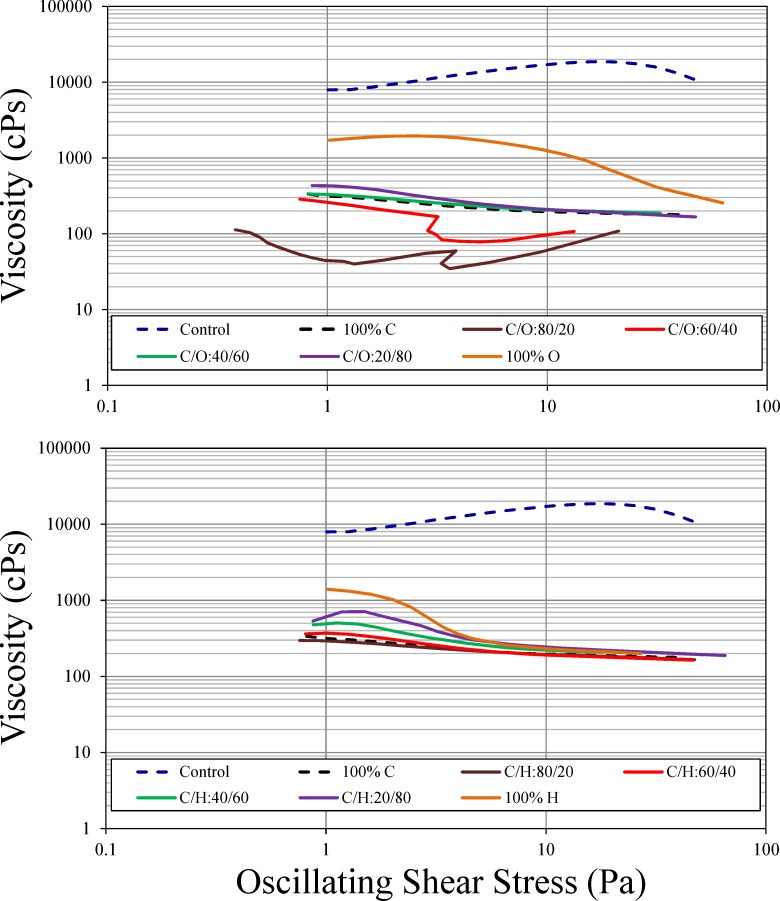
Dynamic viscosity of AWOE (control, 100% dispersant, and as a function of dispersant additive fraction).

Dynamic viscosity of AWOE as a function of shear stress for control, 100% dispersant, and as a function of dispersant additive fractions, is shown in Fig 4. This figure illustrates the non-Newtonian behavior of both untreated and treated AWOE, for both polar additives. Shear stress from wave action is the primary mixing mechanism driving the formation of WOE in natural water bodies, and thus AWOE dynamic viscosity is an acceptable surrogate measure of the change in interfacial viscosity of these emulsions in open water. Untreated AWOE exhibits shear thickening (increasing viscosity with increasing shear stress) while 100% dispersant-treated AWOE exhibits shear thinning (decreasing viscosity with increasing shear stress). AWOE treated with fractional amounts of dispersant and dispersant additive exhibits in most cases shear thinning, with the exception of C/O 80/20 and C/O 60/40 (shear thinning at low shear stress, transitioning to shear thickening at high shear stress; Fig 4). Additionally, for these two C/O fractional treatments, dynamic viscosity is less than that for 100% dispersant-treated AWOE.

The tendency for the viscosity of untreated WOE to increase under increased shear stress provides insight into why these emulsions are often stable in natural aquatic systems. Increasing shear stress tends to disorder the distribution of water droplets in the emulsion causing them to cluster; however, the rigid, mechanically stable asphaltenic films at the water droplet-oil interface resist water droplet coalescence, leading to increased viscosity [32]. Conversely, for AWOE treated with dispersant and modified dispersant, displacement of asphaltenic molecules by other surface active agents in the dispersant or modified dispersant creates more mobile, liquid interfacial films, allowing some water droplet coalescence with a concomitant decrease in viscosity [33]. Fig 4 indicates that both modified dispersant additives, and in general all dispersant/additive fractional amounts, provide similar reductions in AWOE dynamic viscosity. Exceptions to this general observation are 100% 1-octanol (which exhibits shear thinning but is less effective at overall viscosity reduction), and C/O 80/20 and C/O 60/40 fractional treatments (which are more effective at overall viscosity reduction, but exhibit shear thickening at higher shear stress).

**Fig 5:**
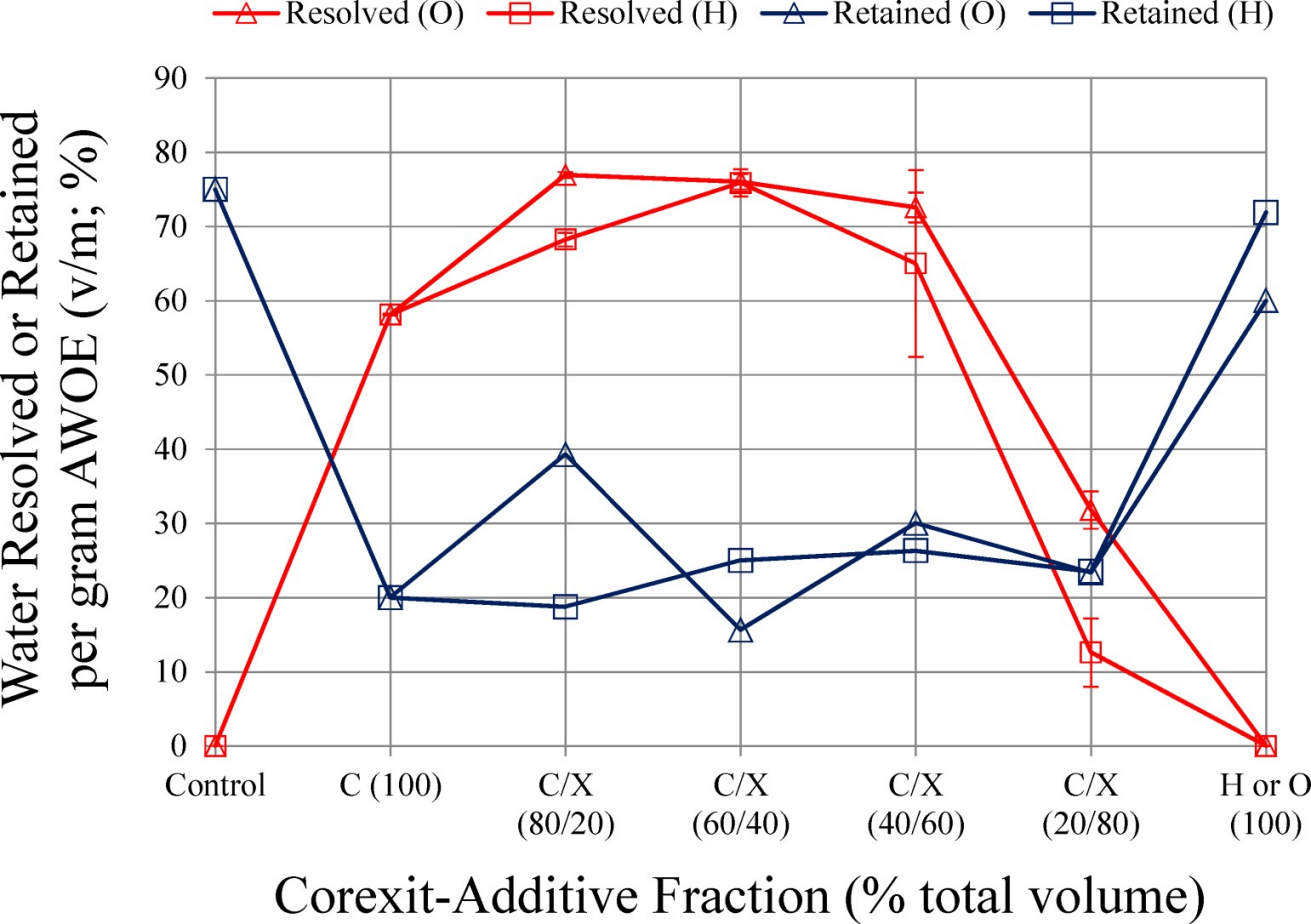
ASW resolved from, and retained by AWOE after treatment with dispersant or modified dispersant.

The amount of ASW resolved from AWOE after treatment with dispersant or modified dispersant is shown in Fig 5. The underlying notion in this experiment is that increased resolved water from AWOE is a measure of the effectiveness of polar additives in modifying the oil-water interfacial film to enhance water coalescence. Fig 5 shows that dispersant alone is reasonably effective at allowing water to resolve from AWOE, although addition of either polar additive (between 20%-60% fraction) enhances this effect. For both polar additives, additive fractions greater than 60% resulted in a macroscopically less viscous emulsion with little to no water resolved. Comparing Fig 5 with Fig 3 suggests this is a consequence of the loss of dispersant-related surfactants and re-accumulation of asphaltenic molecules at the oil-water interface, leading to a transition from a more mobile, liquid state amenable to water coalescence and resolution (the condition for 100% dispersant to less than 60% additive fraction) to a more mechanically stable state resisting water resolution.

**Fig 6:**
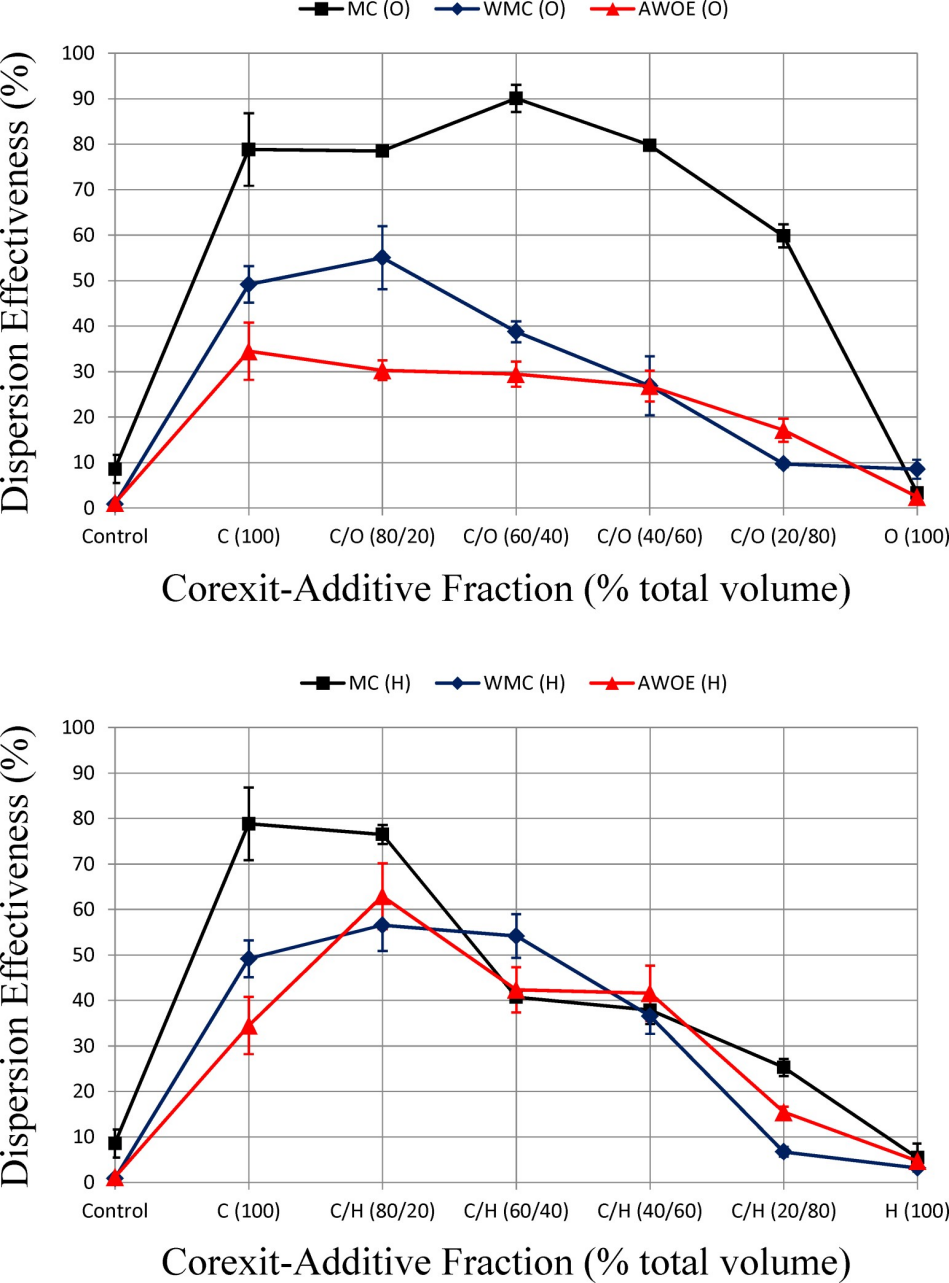
Effectiveness of unmodified and modified dispersant to disperse MC, WMC, and AWOE-associated oil into the water phase.

The effectiveness of unmodified and modified dispersant to disperse oil (MC, WMC, and AWOE-associated oil) into the water phase is shown in Fig 6. For both MC and WMC, dispersion effectiveness is negligibly enhanced (1-octanol) or not enhanced (hexylamine) at low additive fractions compared to 100% Corexit, and dispersant effectiveness is negatively impacted at higher additive fractions compared to 100% Corexit. A similar trend occurs for AWOE and 1-octanol additive fraction. However, dispersion effectiveness increases for AWOE and hexylamine additive fraction between 20%-60%.

### Microscopic analysis

**Fig 7:**
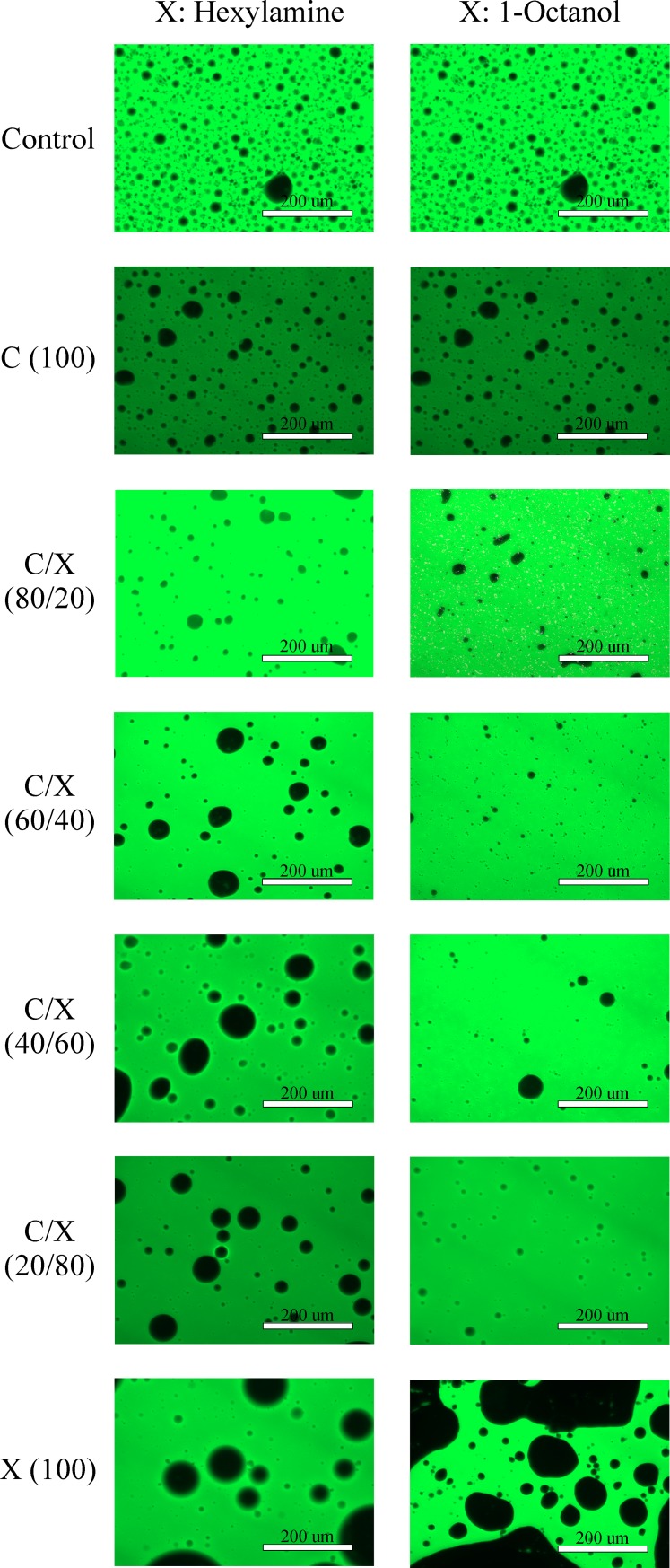
Microscopic images of untreated AWOE, and AWOE treated with dispersant and modified dispersant.

Microscopic images of untreated AWOE, and AWOE treated with dispersant and modified dispersant are shown in Fig 7. AWOE used to create these images was collected following the water resolution experiments. All images are shown in fluorescence mode at the same magnification (200 μm): water appears black, and oil appears green. Duplicate photo-microscopic images at this magnification scale for a given additive treatment level were highly variable in their oil-water content, thus only observations on general trends are warranted. Within these constraints, the images suggest that AWOE treated with 100% dispersant (no additives) results in an increase in water coalescence compared to the untreated (control) AWOE. For both additives, at an additive fraction of 20%, less water is present in the emulsion (corresponding to enhanced water resolution shown in Fig 5). For additive fractions between 40–80%, interpretation of Fig 7 is more ambiguous. For hexylamine, it appears that as the additive fraction increases, water is retained in the emulsion. However, this could also be an artifact of the photo-microscopic method used. Moreover, this effect is less pronounced for 1-octanol in the same additive fraction range, even though one would expect similarities between the additives based on Fig 5. As noted earlier, for both polar additives, as additive fractions increased above 60%, AWOE became (macroscopically) a less viscous emulsion with a corresponding decrease in water resolution. At the 100% additive treatment level, Fig 7 supports results shown in Fig 5 (no water resolved from the emulsion).

### Destabilization of AWOE

Chemical additives are used in petroleum industry to break down W/O emulsions formed during oil production and processing. The principal role of chemical additives is to reduce interfacial viscosity and enhance water coalescence [34]. The two additives selected to modify Corexit (1-octanol and hexylamine) were chosen based on their mechanistic differences in destabilizing W/O emulsions. 1-octanol acts to break down existing intermolecular hydrogen bonds between asphaltene molecules, replacing them with alcohol‐asphaltene hydrogen bonds [22]. Wasan et al. [23] observed similar destabilizing effect for medium‐chain alcohols. Hexylamine acts to disintegrate asphaltenes through interaction between the nitrogen group (base) and the acid groups present in the interfacial film. A consequence of this interaction is that the interfacial film becomes more hydrophilic [22, 24].

Fig 4 shows that the addition of Corexit alone changes the dynamic viscosity of AWOE from shear thickening to shear thinning, and this reduction in interfacial viscosity can be observed in an enhanced ability to resolve water from AWOE (Fig 5), an increase in DE compared to untreated AWOE (Fig 6), and what appears to be limited coalescence of ASW (Fig 7). AWOE treated with modified Corexit (below 60% additive fractions) indicated that for both polar additives used in this study, modest but measurable enhancements in AWOE instability occurred as compared to Corexit alone (indicated by increased water resolution). For additive fractions greater than approximately 60%, AWOE became a more macroscopically fluid emulsion which resisted water resolution and dispersion into ASW.

## Conclusions

This study considered the effectiveness of Corexit 9500A (the most widely available and used oil dispersant world-wide), modified to enhance its polar fraction, in the destabilization of WOE. Results suggest that Corexit modified to include between 20–60% fractional amount of either polar additive (1-octanol or hexylamine) will produce a modest increase in WOE instability, with a Corexit to hexylamine ratio of approximately 80/20 providing the most effective enhanced destabilization (based primarily on increased water resolution and dispersion effectiveness). Although these results are specific to Corexit 9500A, similar modifications to non-Corexit dispersants are likely to produce similar results, since increasing the fraction of polar constituents should affect asphaltene solubility and decrease oil-water interface stability in similar ways. The results presented here support the hypothesis that modifying the fraction of polar constituents in commercial dispersants will increase asphaltene solubility, decrease oil-water interface stability, and enhance WOE instability. Additional studies are needed to optimize this potentially promising strategy for managing WOE in aquatic systems.
